# Swedish version of the multi dimensional health assessment questionnaire – translation and psychometric evaluation

**DOI:** 10.1186/1471-2474-14-178

**Published:** 2013-06-04

**Authors:** Kristina Areskoug Josefsson, Charlotte Ekdahl, Ulf Jakobsson, Gunvor Gard

**Affiliations:** 1 Department of Health Sciences, Division of Physiotherapy, Lund University, Box 157, 221 00, Lund, Sweden; 2 Värnamo Hospital, Samrehab, Värnamo Sjukhus, 331 85, Värnamo, Sweden; 3 Center for Primary Health Care Research, Lund University, CRC, 20205, Malmö, Sweden

**Keywords:** Health assessment, Rheumatoid arthritis, Rehabilitation

## Abstract

**Background:**

Health assessment measurements for patients with Rheumatoid arthritis (RA) have to be meaningful, valid and relevant. A commonly used questionnaire for patients with RA is the Stanford Health Assessment Questionnaire Disability Index (HAQ), which has been available in Swedish since 1988. The HAQ has been revised and improved several times and the latest version is the Multi Dimensional Health Assessment Questionnaire (MDHAQ). The aim of this study was to translate the MDHAQ to Swedish conditions and to test the validity and reliability of this version for persons with RA.

**Methods:**

Translation and adaption of the MDHAQ were performed according to guidelines by Guillemin et al. The translated version was tested for face validity and test-retest in a group of 30 patients with RA. Content validity, criterion validity and internal consistency were tested in a larger study group of 83 patients with RA. Reliability was tested with test-retest and Cronbach´s alpha for internal consistency. Two aspects of validity were explored: content and criterion validity. Content validity was tested with a content validity index.

Criterion validity was tested with concurrent validity by exploring the correlation between the MDHAQ-S and the AIMS2-SF. Floor and ceiling effects were explored.

**Results:**

Test-retest with intra-class correlation coefficient (ICC) gave a coefficient of 0.85 for physical function and 0.79 for psychological properties. Reliability test with Cronbach´s alpha gave an alpha of 0.65 for the psychological dimension and an alpha of 0.88 for the physical dimension of the MDHAQ-S.

The average sum of the content validity index for each item was of the MDHAQ-S was 0.94. The MDHAQ-S had mainly a moderate correlation with the AIMS2-SF, except for the social dimension of the AIMS2-SF, which had a very low correlation with the MDHAQ-S.

**Conclusions:**

The MDHAQ-S was considered to be reliable and valid, but further research is needed concerning sensitivity to change.

## Background

In order to cover aspects of importance to the patients with Rheumatoid Arthritis (RA), the most appropriate outcome measurement for clinical practice must be chosen in the clinical situation. The outcome measurements have to be meaningful, valid and relevant. For patients with RA, questionnaires often are included in standard outcome measurements. Due to the symptoms of RA, physical ability should be measured and a commonly used questionnaire for patients with RA is the Stanford Health Assessment Questionnaire Disability Index (HAQ), which has been available in Swedish since 1988 [1,2]. The HAQ has been used to measure physical function and it has been revised several times. The latest version, the Multi Dimensional Health Assessment Questionnaire (MDHAQ), has a broader perspective and better coverage of the domains in the International Classification of Functioning, Disability and Health (ICF) [3,4], but is not yet available in Swedish. The HAQ mainly concerns the activity domain, which gives a narrower perspective than the MDHAQ [5]. As an example, the MDHAQ has questions concerning fatigue and anxiety, and questions with limited relevance in the HAQ are omitted in the MDHAQ [1,6,7]. The importance of physical activity has increased for patients with RA, and this valuable item is included in the MDHAQ. These differences between the HAQ and the MDHAQ are of major importance, and it is therefore necessary to follow the development of improved health assessment and make the MDHAQ available in different contexts, in order to be able to assess the impact of RA. The MDHAQ has a short completion time compared to other tests, and the data collected are clinically useful [8]. The choice to translate and psychometrically test the MDHAQ under Swedish conditions is due to the increased scope of the MDHAQ and it´s usefulness in clinical practice in comparison with other outcome measurements [9]. The reliability of the original MDHAQ has earlier been tested with test-retest for the first ten items with kappa statistics, giving scores between 0.65 and 0.81 [7], and the translated versions having scores between 0.60 and 0.93 [10,11]. The criterion and construct validity of the MDHAQ has been investigated in earlier studies translating the instrument, with good results [10,11]. The criterion validity of the activities of daily living questions included in the MDHAQ, that derive from the original HAQ, has been tested under Swedish conditions showing that the correlation between the patient’s evaluation of the activity and the therapist’s measure of that activity was 0.91 with a significance of p < 0.0001 [1]. The floor and ceiling effects of the MDHAQ have been reported to be acceptable (i.e. below the recommended cut-off point of 15%) [12,13]. The correlation between the items of the MDHAQ and pain (visual analogue scale), fatigue (visual analogue scale), advanced activities of daily living, anxiety and depression of the Arthritis Impact Measurement Scale (AIMS) showed a range of the Spearman´s rank correlation coefficient between 0.50 and 0.75 [7]. The MDHAQ has been proven suitable for other rheumatic diseases than RA as well, which makes the instrument very useful and provides further reason to have the MDHAQ available in a Swedish version [14]. The MDHAQ [6] is to the best of our knowledge not used in Sweden, since it has not been tested under Swedish conditions and/or translated into Swedish.

### Aim

The aim of the study was to test the reliability, face validity, content validity and criterion validity of a Swedish version of the MDHAQ.

## Method

### Sample

There were two groups of participants included in the study, each group was used for different tests; a reliability test group and a validity test group. The participants in the study were diagnosed with RA and lived in the south of Sweden. The participants from a rehabilitation clinic were included consecutively into a smaller test group testing reliability of the MDHAQ-S. The participants from the rheumatology clinic were included consecutively in the larger test group, testing validity of the MDHAQ-S. Data were collected with questionnaires. The participants in both groups were included in the study consecutively. Written and informed consent for participating in the study was obtained from the participants. Persons who did not understand Swedish were excluded. Data of persons not willing to participate in the study were not collected. A registered physiotherapist or a registered nurse at the rehabilitation/rheumatological clinic assessed if the participant had sufficient knowledge of Swedish to be able to answer the questionnaires.

#### Inclusion in reliability test group

The reliability test group consisted of 30 persons with RA (diagnosed by a rheumatologist), aged > =18 yrs, registered at a rehabilitation clinic in the south of Sweden. Test-retest was to be performed by this group.

#### Inclusion in the validity test group

The validity test group consisted of 100 persons with RA (diagnosed by a rheumatologist), > = 18 yrs, registered at a clinic for rheumatology in the south of Sweden.

#### Instruments used

The MDHAQ-S and Arthritis Impact Measurement Scale 2– Short Form (AIMS2-SF) were used in the study. The AIMS2-SF [15] was used to measure the concurrent validity of the MDHAQ-S. The age of the participants was not included in the questionnaires.

#### MDHAQ-S

The MDHAQ-S consists of the following parts: physical function, psychological status, pain, global health, fatigue, morning stiffness, and exercise habits, and includes Routine Assessment of Patient Index Data 3 (RAPID3) and Rheumatoid Arthritis Disease Activity Index (RADAI) self report joint count as well as a symptoms list and recent medical history [9,14]. The MDHAQ-S is estimated to be performed in five minutes. The first section, “physical function”, of the MDHAQ-S (question1. a-j) includes ten activities of daily living scored 0–3 (0 = “without any difficulty”, 1 = “with some difficulty”, 2 = “with much difficulty”, and 3 = “unable to do.”). The sum of the answers is divided by three giving a score between 0 and 10. The questions concerning psychological status (question1.k-m) are scored 0 = “without any difficulty”, 1.1 = “with some difficulty”, 2.2 = “with much difficulty”, and 3.3 = “unable to do.” The sums are added to a total sum of 0–9.9. The MDHAQ-S includes visual analogue scales with 21 circles measuring pain, global health and fatigue with a total score of 0–10 in 0.5 units. The review of symptoms (question 5) is a checklist of symptoms where the checked boxes are counted. Morning stiffness (question 6) is rated by yes or no and the amount of time in minutes. Change in status (question 7) is scored: 1 = Much better, 2 = Better, 3 = Same, 4 = Worse, 5 = Much worse. Exercise frequency (question 8) is scored 3 = 3 or more times a week, 2 = 1–2 times per week, 1 = 1–2 times per month, 0 = Do not exercise regularly, 9 = Cannot exercise due to disability/ handicap. The included RADAI consists of eight joints or joint groups scored 0, 1, 2 or 3. The RADAI scores are used in the RAPID3 (0–30 scale), which include four categories: High severity >12, Moderate severity = 6.1-12, Low severity = 3.1-6, and Remission < =3. The included RAPID3 and the RADAI have high reliability [16]. The RADAI is a valid instrument [17,18], and patient reported tender joint count has a moderate to marked correlation to assessment by health professionals [19].

The MDHAQ-S also includes questions about recent medical history, which are not scored.

#### AIMS2-SF

The AIMS2-SF is a self administered questionnaire with 26 items covering the ICF components activity limitations and participation restrictions [15] within five domains: physical function, symptoms, mood, social function and role function. Each item is answered using a five-point scale. Higher scores indicate higher level of impairment. The time to perform the AIMS2-SF is estimated to be ten minutes. The Arthritis Impact Measurement Scale (AIMS) was developed to assess outcome of healthcare for patients with RA [20] and has been further developed and shortened to the Arthritis Impact Measurement Scale 2-Short Form (AIMS2-SF) [15]. The AIMS measures the individual´s functional, social, emotional and physical status. The AIMS-SF2 has been compared to the HAQ and been found to have better sensitivity to change than the HAQ [21]. This makes the AIMS2-SF a relevant instrument for use for comparison with MDHAQ-S, and the instrument is relevant in terms of its contents to persons with RA [1]. Both the HAQ and the AIMS 2 are valid and reliable instruments in Swedish [1,22]. The AIMS has also been used to test the validity of other outcome measurement tools for patients with RA [23].

### Translation and adaptation of the MDHAQ-S

The adaption of the MDHAQ R808 [24] to a Swedish version (MDHAQ-S) was performed according to the guidelines by Guillemin et al. [25,26], which include the following steps:

1. Translation of the MDHAQ by two independent qualified translators.

2. Synthesize translations, which meant to compare the translated versions in order to achieve coherence in translations. This was performed by the authors.

3. Back-translation by two independent back-translators.

4. Committee review by a multi-professional committee. The committee was a rheumatologic team consisting of a rheumatologist, physiotherapist, counselor, occupational therapist, nurse and one of the researchers.

5. Thirty patients testing the questionnaire with an interviewer present. The interviewer can be used as an explanatory source if needed. The interviewer observes whether there are problems reading or responding to the questionnaire and asks about the ease of completion of the questionnaire. This step is revised from the original guidelines by Guillemin and follows the protocol by Hedin et al. [27].

### Reliability of the MDHAQ-S

Reliability was tested with test-retest with a one-week interval. The test-retest was performed at a rehabilitation clinic in a county in the south of Sweden. Reliability was also assessed with Cronbach´s alpha for internal consistency.

### Validity of the MDHAQ-S

The floor and ceiling effects of the MDHAQ-S were measured in the validity test group. Floor effects were considered to be present if ≥15% scored an item as 0 (lowest possible score) and ceiling effects were considered to be present if ≥ 15% scored an item as 3 (highest possible score) on the MDHAQ-S. The content validity of the MDHAQ-S was tested by the reliability test group. The relevance of each question included was assessed with a content validity index, CVI, on a four-point scale (1- extremely relevant, 2 – quite relevant, 3 – slightly relevant and 4 – not relevant) [28]. The scale was dichotomized by putting extremely relevant/quite relevant (1 & 2) into one group and slightly relevant/not relevant (3 & 4) into one group. This test was performed when the original HAQ was adapted to Swedish [1], and comparisons of the relevance of questions existing in both the HAQ and the MDHAQ-S were made. The participants were asked to add additional important questions that they felt were lacking in the instrument.

Face validity was performed within a group of professional experts as well as by the participants by their rating of the relevance of the questions of the MDHAQ-S. The group of professional experts consisted of a rheumatologist, physiotherapist, counselor, occupational therapist, nurse and one of the researchers.

Criterion validity by measuring concurrent validity was tested by asking the respondents to complete the MDHAQ-S and the AIMS2-SF and testing the results for correlation. The MDHAQ-S was tested against the Arthritis Impact Measurement Scale 2- Short Form (AIMS2-SF) for correlation. The AIMS2-SF measures the individual´s functional, social, emotional and physical status. The AIMS2-SF is a shorter and less time consuming version of the Arthritis Impact Measurement Scale −2 (AIMS2) with similar psychometric properties, convergent validity content validity, reliability and sensitivity to change [15,21,29]. The AIMS2-SF is easier for the patients to administer since it consists of fewer questions, 26 instead of 57. In order to achieve the best possible symptom agreement concerning arthritis pain between AIMS2 and AIMS2-SF, item 42 was replaced with item 38 in AIMS2-SF, which has been recommended in earlier studies [21,30]. The AIMS2-SF is a relevant instrument for use for comparison with MDHAQ-S due to the instrument´s relevance of its contents to persons with RA and since the AIMS was used in the comparison with the HAQ when it was adapted to Swedish conditions [1]. The AIMS has also been used to test the validity of other outcome measurement tools for patients with RA [23], and both the AIMS and the AIMS 2 are valid and reliable instruments in Swedish [1,22].

### Procedures

The reliability test group: The participants answered the questionnaire twice, once at the visit to the clinic and a second time one week after the visit. The participants answered the first questionnaire at a visit to the clinic with a physiotherapist present. The questionnaires were handed out and collected by the physiotherapist at the clinic.

The validity test group: The participants answered the questionnaires at a visit to the clinic. The questionnaires were handed out and collected by the nurse at the clinic.

### Data analysis

The reliability of the Swedish version was assessed with Cronbach´s alpha for internal consistency and test-retest for reproducibility with weighted kappa statistics and intra-class correlation. A high alpha, over 0.7, indicates that the items are adequately inter related [31]. The test-retest measure is used to estimate the reproducibility over time when no change is estimated to have taken place. The kappa values were considered to show excellent reliability if they were >0.75, fair to good reliability for values ranging between 0.4 and 0.75 and moderate to poor agreement for values <0.4 [32]. The floor and ceiling effects of the MDHAQ were analyzed. Content validity was tested with the content validity index (CVI). The items were considered to be relevant if the item-level CVI was >0.78 per item and the MDHAQ-S was considered to be relevant if the average of the sum of the content validity index for each item was > 0.90 [28,33]. Concurrent validity was estimated by assessing the level of association between scores on the MDHAQ and the AIMS2-SF, with Spearman rank order correlation. Questions 5, 6 and 7 of the MDHAQ-S were not tested for correlation, since they were not considered by the authors to be relevant to be compared with AIMS2-SF. The correlations were measured comparing the total scores for each dimension of AIMS2-SF with total scores of the included dimensions of the MDHAQ-S.

The level of significance was set at p < 0.05. Data analyses were performed by SPSS 18.0 and VassarStats: website for statistical computation.

### Ethics approval

The study was approved by the regional ethics committee in Linköping (d.no: 2011/142-31).

## Results

The translation and adaption process of the MDHAQ led to the removal of two items as they were not applicable to Swedish context, “ethnic group” and “change of medical insurance” – part of question 10 (”Over the last 6 months you have had…”). Questions concerning ethnic group are unusual in Swedish health care questionnaires and was excluded according to the recommendation of the multi professional committee review, in step 3 of translational process. The other parts of the original MDHAQ were kept, and no additional items were included in the Swedish version. None of the participants in the reliability test group wanted to add or withdraw items to/from the MDHAQ-S and they considered to the MDHAQ-S to be comprehensible and acceptable.

There were 100 patients who were invited to participate in the validity test group and 83 persons agreed to participate. There were 58 (70%) women and 19 men (23%) who participated. Six persons did not describe their gender. The results for each item with mean, standard deviation, range, response rate, floor effects and ceiling effects are presented in Table 1.

**Table 1:** Characteristics for the MDHAQ-S items and scores

**Item/scale score**	**Mean (SD)**	**Range**	**Response rate%**	**Floor,%**^**1**^	**Ceiling,%**^**2**^
1a. Dressing yourself, including tying shoelaces and doing buttons?	0.49 (0.59)	0-3	99%		
1b. Get in or out of bed?	0.28 (0.45)	0-3	99%		
1c. Lift a full cup or glass to your mouth?	0.29 (0.48)	0-3	99%		
1d. Walk outdoors on flat ground?	0.27 (0.52)	0-3	99%		
1e. Wash and dry your entire body?	0.39 (0.52)	0-3	99%		
1f. Bend down to pick up clothing from the floor?	0.48 (0.65)	0-3	99%		
1g. Turn regular faucets on and off	0.51 (0.74)	0-3	99%		
1h. Get in or out of a car, bus, train, or airplane?	0.46 (0.61)	0-3	98%		
1i. Walk 3 km?	0.90 (1.00)	0-3	98%		
1j. Participate in sports and games as you would like	1.35 (1.03)	0-3	94%		
Mean 10-item physical function score	1.76(1.56)	0-3	93%	14,5%	0%
1k. Get a good night’s sleep?	1.05 (0.86)	0-3	96%		
1l. Deal with the feelings of anxiety or being nervous	0.42 (0.52)	0-3	94%		
1m. Deal with the feelings of depression or feeling blue?	0.46 (0.55)	0-3	95%		
Mean 3 item psychological score	1.96 (1.52)	0-3	93%	21,7%	0%
2. How much pain have you had because of your condition over the past week?	4.03 (2.31)	0-10	98%	6%	0%
3. Amount of pain in joints	13.12 (7.94)	0-54	99%	3,6%	0%
4. Considering all the ways in which illness and health conditions may affect you at this time, please indicate below how you are doing	3.69 (2.30)	0-10	98%	6%	0%
5. Amount of symptoms	10.05 (6.18)	0-60	99%	3,6%	0%
6. Morning stiffness, yes-no	1.32 (0.47)	1-2 (yes/no)	95%		
7. How do you feel today compared to one week ago?	2.59 (0.76)	1-5	98%	7,2%	1,2%
8. How often do you exercise aerobically?	1.36 (1.25)	0-4	93%	38,6%	21,7%
9. How much of a problem has unusual fatigue or tiredness been to you over the last week?	4.30 (2.79)	0-10	94%	8,4%	2,4%
10. Amount of other events	1.37 (1.32)	0-12	90%	28,9%	0%

^1^Worst possible value of the item or minimum total value of the scale. ^2^Best possible value of the item or maximum total value of the scale.

### Reliability

The reliability test with Cronbach´s alpha gave an alpha of 0.65 for the psychological dimension of the MDHAQ-S and an alpha of 0.88 for the physical dimension of the MDHAQ-S. Testing item total correlation showed that, if the item concerning sleep was removed from the psychological dimension, the Cronbach´s alpha increased to 0.91. In the physical dimension of the MDHAQ-S, Cronbach´s alpha remained stable when testing item total correlation for the items included (variance 0.86-0.87).

The reliability test group had 27 fully responded questionnaires which were used for the calculations. Test-retest was performed in two ways. The items for physical function item 1a- 1j were first summarized to one score and the items for psychological function were also summarized to one score. Test-retest with intra-class correlation coefficient (ICC) gave a coefficient of 0.85 for physical function and 0.79 for psychological properties, which showed a good reliability of the MDHAQ-S for the functional and the psychological properties.

The second analysis of test-retest was performed for all items in the MDHAQ-S separately. The kappa statistics of the items 1a-1 m showed a range between 0.35 and 0.82. The items with the highest scores were “Deal with feelings of depression or feeling blue?” (Kw = 0.82) and “Turn regular faucets on and off?” (Kw = 0.72), both showing excellent reliability. The items with the lowest scores were “walk three kilometers, if you wish” (Kw = 0.35), “Deal with feelings of anxiety or being nervous?” (Kw = 0.39) and these two items show poor reliability. The other items in the first question have kappa values varying between 0.46 and 0.73, which is considered to be fair to good reliability.

Items 2–5 and 9–10 had an ICC of 0.75-0.86, which indicates very good reliability (items 5 and 10 concerning the amount of difficulties experienced). Items 6–8 were measured with kappa statistics and showed acceptable to very good results. Item 6 had a kappa of 0.51. This item also included a question concerning the duration of morning stiffness, which had an ICC of 0.28. Item 7, “How do you feel today compared to one week ago?”, had a kappa of 0.41. Item 8, “How often do you exercise aerobically (sweating, increased heart rate, shortness of breath) for at least one-half hour (30 minutes)?”, had a kappa of 0.95.

### Validity

The floor and ceiling effects of the MDHAQ-S are below the cut-off point of 15% for the physical dimension, showing that RA has had a negative effect on their physical capacity. The psychological dimension has a floor effect of 21.7% and a ceiling effect of 0%, which means that the participants has scored low effect of RA on their psychological function. There were floor and ceiling effects in question 8 concerning exercise habits and floor effects for question 10 (Table 1). Question 10 concerned changes in their medical history, such as having experienced a medical trauma, and in their social life, for example changed their medical status, during the last six months. The floor effect of this item shows that the participants had quite stable medical and social status.

The items were considered to be relevant if the content validity index was >0.78. Item 10 in the MDHAQ-S had an item-level CVI of 0.75. The range of the item-level CVI of the other items in the MDHAQ-S was 0.89-1.00, which indicates that those questions in the MDHAQ- S are highly relevant for persons with RA. The MDHAQ-S was considered to be relevant since the average of the sum of the content validity index for each item was 0.94.

None of the items in the MDHAQ-S and the AIMS2-SF had a very high correlation (Table 2). There were correlations for some of the items, with item 2 (pain) of the MDHAQ-S and the symptoms dimension of the AIMS2SF having a high correlation (r = 0.77). Item 8 (level of physical exercise) had a very low correlation with all items in the MDHAQ-S and the AIMS2-SF.

**Table 2:** Correlation between the MDHAQ-S and the AIMS2-SF

	**MD1 sum physical**	**MD 1Sum psychological**	**MD2**	**MD3 total sum**	**MD4**	**MD8**	**MD9**	**AIMS2 physical**	**AIMS2 symptoms**	**AIMS2 mood**	**AIMS2 social**	**AIMS2 role**	**AIMS2 total**
MD1 Sum	-												
Physical													
MD1 Sum	**0.403**	-											
MD2	**0.617**	**0.357**	-										
MD3 Total sum	**0.549**	**0.419**	**0.487**	-									
MD4	**0.593**	**0.431**	**0.708**	**0.514**	-								
MD8	**−0.240**	−0.127	−0.128	−0.031	−0.204	-							
MD9	**0.370**	**0.534**	**0.471**	**0.431**	**0.468**	−0.075	-						
AIMS2-SF	**0.442**	0.106	**0.284**	0.179	0.210	−0.141	0.136	-					
Physical													
AIMS2-S	**0.646**	**0.355**	**0.767**	**0.578**	**0.582**	−0.064	0.384	0.269	-				
Symptoms													
AIMS2-SF	**0.252**	**0.565**	**0.297**	**0.427**	**0.416**	−0.072	0.445	0.055	**0.339**	-			
Mood													
AIMS2-SF	0.001	0.154	0.022	−0.023	0.148	−0.046	0.015	0.199	0.075	**0.392**	-		
Social													
AIMS2-SF	**0.435**	0.249	**0.413**	**0.330**	**0.436**	0.012	0.212	0.077	**0.339**	0.290	0.162	-	
Role													
AIMS2-SF	**0.528**	0.264	0.283	0.269	0.293	−0.177	0.206	**0.854**	**0.447**	**0.384**	**0.602**	**0.360**	-
Total													

Significant (p ≤ 0.05) correlations are in bold in the correlation matrix.

## Discussion

The results of this study indicate that the MDHAQ-S is a reliable and valid instrument that can be of use in rheumatologic clinical care among Swedish speaking patients with RA. The choice of forward-backward translation according to the guidelines of Guillemin et al. [25,26] can be discussed, since a comparison between forward-backward translation and dual-panel methodologies has shown differences [34]. The fifth step in the translational process was therefore changed, as recommended by Hedin et al. [27], in order to bring out the opinions of the patients for whom this test is intended to be used for in the future.

### Reliability

The size of the reliability test group was similar to the group size in the reliability test of the Finnish version of the MDHAQ [10]. In the Finnish version, only item 1a-m (for item description, see Table 1) was analyzed with test-retest and the scores were summarized with one score for physical function and one for psychological properties. The ICC in the Finnish study was 0.94 for physical function and 0.84 for psychological properties [10], which shows a better reliability than the results in our study. The Arabic version of the MDHAQ also had better ICC results, 0.99 for physical function and 0.65 for the psychological dimension [35]. However, they had only 48–96 hours between the test and the retest, compared to one week in this study, and there were some differences in the questions that were included, which might have affected the results. The Korean test-retest of the MDHAQ tested reliability item

by item [11], however. The results of the Korean version with kappa statistics ranged from 0.60-0.76 (p < 0.001), which is considered good reliability, and the results in our study ranged from 0.39-0.82, which indicates a more varied reliability for the items in the first question concerning physical and psychological function in the MDHAQ-S. When using the recommended limits given by Kirkwood & Sterne [32], the MDHAQ-S shows varying results for reliability, but the majority of the items 1a-m have good reliability. The differences in the kappa statistics can be the reason why the ICC in our study was lower than in the Finnish study. The original MDHAQ had kappa scores of 0.65-0.81, p < 0.001, for the items in the first question concerning physical and psychological function [7], but there were differences in some of the items compared to the current MDHAQ. The MDHAQ-S is reliable, but the results show that the different translated versions of the MDHAQ vary in reliability in different contexts.

Cronbach’s alpha of the original MDHAQ was 0.92 [6], which is higher than the scores of the translated versions. The Cronbach´s alpha of the psychological dimension of the Korean version of the MDHAQ was 0.89, and the Cronbach’s alpha for the physical function was 0.89 [11]. The results of our study are similar to that of the Finnish version, which had a Cronbach’s alpha of 0.66 for the psychological function and 0.92 for the physical function [10]. In the Finnish version, the item correlation of the sleep question was clearly lower than the other psychological items [10], and this study shows the same result. Internal consistency for AIMS2SF and MDHAQ was discussed as being indeterminate in a study by Oude Voshar et al. (2011) since the Cronbach’s alpha has been performed for the complete dimension of physical function, while it may be argued that it is more appropriate to divide this dimension further [12].

The floor effect of the psychological dimension of the MDHAQ-S is of interest for further research since an earlier study of the MDHAQ has shown no floor effect [12]. It is however unclear from that study whether all of the MDHAQ has been included or if only the first items in the physical and psychological dimension were included in the test. The Finnish version of the MDHAQ did not show a floor effect of the psychological dimension, and neither did the Korean version nor the original version of the MDHAQ [3,10,11]. The floor effects and ceiling effects for question 8 (“How often do you exercise aerobically (sweating, increased heart rate, shortness of breath) for at least one half hour (30 minutes)?”) could imply that this question needs to be further explored. Due to the strong recommendations of physical exercise for persons with RA [36,37], a more specific instrument for physical exercise habits might be needed as a complement to the MDHAQ-S. Question 10 showed floor effects and, considering the type of question (changes in lifestyle, accidents etc. over the last 6 months), this is difficult to avoid. Overall, the floor and ceiling effects of the MDHAQ- S are acceptable, even if some items need further exploration.

### Validity

The MDHAQ-S had very good content validity. Concurrent and criterion validity of the MDHAQ is good even if there are some questions that might need further development. The AIMS, the AIMS2 and the AIMS2-SF have been used in several other studies for comparison of patient reported outcome measurements [10,11,21,38]. The AIMS2-SF covers some of the items of the MDHAQ-S, but there are differences between the instruments in their coverage. In the Finnish version of the MDHAQ there was a high correlation between the physical dimension of AIMS2 and the physical component of the MDHAQ, but only a low correlation was found in our study [10]. The other correlation coefficients showed similarities between our study and the Finnish study.

The social dimension of the AIMS2-SF showed a very low correlation with the items in the MDHAQ-S. This dimension might be considered difficult to use for comparison due to the changes that have taken place in people´s social life in Western countries, since it also had a low correlation in the Finnish study of MDHAQ [10]. Today´s social life does not demand physical capacities in the same way as before, since a great deal of social life is held over the internet or by phone. This must be considered in future studies.

Some of the patients in the reliability test group found it difficult to rate the relevance of the questions of the MDHAQ, since they thought that the relevance could differ during the years with the disease. The question about the amount of aerobic exercise (question 8) is difficult to compare with the other questions in the MDHAQ-S since there are so many things that influence exercise habits and all of those are not correlated to disease [39], which can be a reason for the floor and ceiling effects of this question. Motivation, time and other factors also play an important role. The importance of regular exercise for persons with RA is however a strong reason why this question should still be included in the MDHAQ [40]. The question about exercise habits had excellent agreement in test-retest, but this could be due to the fact that it takes a long time to change exercise habits. The question should also probably be reformulated and include more steps in order to be sensitive to change. The validity of the MDHAQ is good, but further research to compare the MDHAQ-S with items of social life would be beneficial.

### Further research

There are several areas of interest for further research concerning the MDHAQ-S, such as the sensitivity to change of the MDHAQ, floor effects of the psychological dimension, correlation between items of social life and the MDHAQ-S and further development of the question concerning exercise habits. The MDHAQ-S should also be further investigated in different phases of RA, in order to find out whether the relevance of the MDHAQ-S is similar during the duration of the disease. In this study, the number of years with the disease and the medications were not described by the participants; they could therefore be in different phases of their disease.

Health literacy has been shown to have connections with lower physical function measured with the MDHAQ and it would be of interest to investigate this issue in a Swedish context [41]. Since several of the participants in the study have had RA for a long time, they have encountered this type of questionnaire earlier and may therefore have found the questionnaire easier to fill in than if they had been newly diagnosed and unfamiliar with this type of questionnaire. The level of education is included in the MDHAQ-S but, since it has no correlation with health literacy, it may be possible to remove this item from MDHAQ-S in the future [41].

## Conclusion

The MDHAQ-S has good reliability and validity and can be of use in clinical care for patients with RA, even if there are items that should be further developed to improve the MDHAQ-S. Further research is recommended concerning sensitivity to change.

## Abbreviations

AIMS: Arthritis Impact Measurement Scale; AIMS 2: Arthritis Impact Measurement Scale 2; AIMS2-SF: Arthritis Impact Measurement Scale 2- Short Form; CVI: Content validity index; HAQ: Stanford Health Assessment Questionnaire Disability Index; ICC: Intra-class correlation coefficient; ICF: International Classification of Functioning, Disability and Health; MDHAQ: Multi Dimensional Health Assessment Questionnaire; MDHAQ-S: Multi Dimensional Health Assessment Questionnaire, Swedish version; RAPID3: Routine Assessment of Patient Index Data 3; RADAI: Rheumatoid Arthritis Disease Activity Index.

## Competing interests

The authors declare that they have no competing interests.

## Authors’ contributions

The planning of the study was performed by KAJ, CE and GG. KAJ performed the data collection. The data analysis was performed by KAJ, GG, UJ. KAJ drafted the manuscript and all authors reviewed and approved the final manuscript.

## Pre-publication history

The pre-publication history for this paper can be accessed here:

http://www.biomedcentral.com/1471-2474/14/178/prepub
